# Translation of Fundus Image to Fundus Fluorescein Angiography Boosted by Structure Self-Supervised Representation Cycle Learning

**DOI:** 10.2174/0115734056374967250704090646

**Published:** 2025-07-18

**Authors:** Xiaopeng Wang, Chaoyong Liu, Ruotong Mu, Yi Chen, Di Gong, Qiang Yang, Qiang Liu

**Affiliations:** 1 Academy of Artificial Intelligence, Beijing Institute of Petrochemical Technology, Beijing 102617, China; 2 Department of Ophthalmology, China-Japan Friendship Hospital, Beijing 100029, China

**Keywords:** Medical image, Image analysis, Image translation, Fundus fluorescein angiography generation, Self-supervised learning, Cycle learning

## Abstract

**Introduction:**

Fundus fluorescein angiography captures detailed images of fundus vasculature, enabling precise disease assessment. Translating fundus images to fundus fluorescein angiography images can assist patients unable to use contrast agents due to physical constraints, facilitating disease analysis. Previous studies on this translation task were limited by the use of only 17 image pairs for training, potentially restricting model performance.

**Methods:**

Image pairs were collected from patients through a collaborating hospital to create a larger dataset. A fundus image to fundus fluorescein angiography translation model was developed using structure self-supervised representation cycle learning. This model focuses on vascular structures for self-supervised learning, incorporates an auxiliary branch, and utilizes cycle learning to enhance the main training pipeline.

**Results:**

Comparative evaluations on the test set demonstrate superior performance of the proposed model, with significantly improved Fréchet inception distance and kernel inception distance scores. Additionally, generalization experiments conducted on public datasets further confirm the model's advantages in various evaluation metrics.

**Discussion:**

The enhanced performance of the proposed model can be attributed to the larger dataset and the novel structure self-supervised cycle learning approach, which effectively captures vascular details critical for accurate translation. The model's robust generalization across public datasets suggests its potential applicability in diverse clinical settings. However, challenges such as computational complexity and the need for further validation in real-world scenarios warrant additional investigation to ensure scalability and clinical reliability.

**Conclusion:**

The proposed model effectively translates fundus images to fundus fluorescein angiography images, overcoming limitations of small datasets in previous studies. This approach demonstrates strong generalization capabilities, highlighting its potential to aid in large-scale disease analysis and patient care.

## 1 INTRODUCTION

Fundus image (FI) is an imaging technique used to record the structures of the fundus, primarily utilized in ophthalmic clinical diagnosis and screening [1-3].

FI plays a crucial role in the early diagnosis and treatment of various ophthalmic diseases, such as diabetic retinopathy, macular degeneration, glaucoma, and myopia. Due to its non-invasive nature and high resolution, FI has become an indispensable tool in clinical ophthalmic practice [4, 5].

Fundus fluorescein angiography (FFA) is a medical examination technique used to assess the detailed structure and function of the fundus vascular system [6-8]. This examination is performed by ophthalmic professionals and is of significant clinical importance for diagnosing and monitoring diseases involving the retina and choroid, particularly diabetic retinopathy, macular degeneration, and choroiditis [9, 10].

Although FFA provides critical information about vascular structure and function, it requires the injection of fluorescein into the body, which carries potential risks, including allergic reactions in some individuals [11-13]. Therefore, physicians must carefully weigh the necessity of this examination before proceeding.

The translation of FI into FFA offers several compelling advantages. This innovative approach enables the generation of contrast-like visualizations through a non-invasive process, potentially reducing the need for invasive procedures such as contrast agent injections, which may carry associated discomfort or risks for patients. By leveraging existing color images, this technique eliminates the reliance on specialized equipment or intricate protocols, facilitating its integration into routine workflows with enhanced efficiency and cost-effectiveness. Furthermore, it provides ophthalmologists with a novel tool to extract detailed visual information from standard color fundus photographs, potentially enhancing the detection and analysis of retinal features critical for disease screening and diagnosis. While further validation is required to establish its clinical robustness, this method holds significant promise for advancing non-invasive, efficient, and economical imaging solutions in ophthalmology.

Image translation [14-18] is a computer vision technique aimed at converting one type of image into another. This process typically involves using deep learning [19-22] models to learn the mapping relationships between different image domains [23, 24]. Pix2pix [25, 26] used a conditional GAN that conditions both the generator and discriminator on the input image. This configuration ensures that the generated output is directly influenced by the input. CutGAN [27] does not rely on cycle consistency loss; instead, it uses contrastive learning to achieve translation between image domains, simplifying the training process. StarGAN [28-30] decomposed the image translation task to handle image-to-image translation across multiple domains. StillGAN [31] introduced local structure and illumination constraints to the GAN network, learning both global features and local details of medical images.

FI to FFA is a specific instance of medical image translation [32-37]. Fundus2Angio [38] proposed a coarse-to-fine image translation method, generating fluorescein angiography at resolutions of 256 and 512, achieving favorable translation results on the validation set. Attention2AngioGAN [39] adjusted the model framework from previous work and introduced an additional attention mechanism, resulting in improved performance on the validation set. VTGAN [40] adopted the coarse-to-fine model framework and introduced additional transformer blocks at the discriminator for image classification training, enhancing validation accuracy. Li *et al*. [41] argued that the importance of image pixels varies and introduced an additional loss function to constrain image generation based on the GAN network, achieving good results. The patch-based training method has also been proven applicable to the generation of multi-frame high-resolution FA [34]. UWAT-GAN [42] focused on ultra-wide-angle tasks, training both the full image and patch images separately, while UWAFA-GAN [43] further introduced a registration operation, making the model more focused on generating paired images.

However, previous studies have faced the issue of severely limited or unavailable training data, with only 17 image pairs available for training [38-40], which may not accurately model the relationships between FI and FFA in real-world scenarios. Due to the scarcity of test data, experimental validation of model effectiveness is also insufficient. This limitation prevents the inference model from being truly applicable to practical medical settings.

Fundus images from 331 patients were collected for this study at a local hospital, screening and organizing a training dataset of 433 image pairs. We proposed a model for generating fluorescein angiography images from fundus images. Specifically, we introduced a self-supervised representation cycle learning method, which leverages the structural information within the images to guide model training. An auxiliary learning-based branch is incorporated, along with cycle learning, to further enhance the primary model's performance in the image generation task. The code is available at: https://github.com/poonker/fi2ffa.

## 2 MATERIALS AND METHODS

This study decouples the research objective into image structure extraction and style generation. A self-supervised representation learning method is introduced to enhance the model's focus on image structures. An auxiliary learning approach is employed to establish branch training tasks, improving the model's performance on the main task. Additionally, cycle learning guides the model to focus on the intrinsic style feature of the images.

This section introduces the overall methodology: Section 2.1 outlines the model workflow, Section 2.2 details the model framework, Section 2.3 describes an auxiliary learning approach, Section 2.4 presents a structure self-supervised representation learning method, Section 2.5 explains cycle learning, and Section 2.6 introduces the loss functions involved in computation.

### 2.1 Model Workflow

FI to FFA involves four image domains. Domain FI and Domain FFA represent the domains of the fundus image dataset and the fluorescein angiography dataset, respectively. Domain fundus image’s structure (FIS) and domain fundus fluorescein angiography structure (FFAS) represent the domains of the corresponding images obtained through the structure extraction algorithm.

As shown in Fig. (**1**), during the training process, for any image *x* the model *NetG* translates it into the corresponding image *y’*. The fundus image structure *x’_s_* is then input into the next model to generate the cyclically translated fundus image *x”* and the fundus structure image *x”s*. For *y*’ and *x’_s_*, the discriminator networks (dark blue), along with the corresponding norm loss, are used to constrain the model's output. For *x*” and *x”s*, only the norm loss is used to constrain the model training.

Similarly, the same training process is applied to FFA. For any image *y*, the model translates it into the corresponding image *x*’ and the fluorescein angiography structure *y*’_s_ is then input into the next model to generate the cyclically translated fluorescein angiography image *y*” and the structure image *y*”_s_.

### 2.2 Model Framework

Generative Adversarial Networks (GAN) [44] consist of two neural networks—a generator and a discriminator—trained in an adversarial manner. The generator aims to synthesize realistic images, while the discriminator distinguishes between real and generated samples. Through this competitive process, the generator gradually improves, producing high-quality outputs. GANs have been widely applied in medical imaging for tasks such as image synthesis, domain adaptation, and enhancement. In this study, modular incremental modifications are made based on the GAN network framework.

The U-shaped structure is employed as the main part of the generator, a framework that has gained widespread attention due to its use in U-Net [45]. This framework is divided into an encoder and a decoder. The encoder is responsible for compressing information and extracting features, while the decoder decompresses the information and reconstructs the image. The encoder extracts hierarchical features through convolutional layers followed by 2 × 2 max-pooling, progressively reducing spatial dimensions while increasing feature channels. The decoder mirrors the encoder, utilizing transposed convolution operations and skip connections to reconstruct the spatial resolution of the feature maps. These skip connections link the encoder and decoder layers of corresponding resolutions, ensuring the preservation of fine-grained spatial details. In this study, in the decoder section, an inherent design framework is repeatedly used to establish additional mirror branches. As shown in Fig. (**1**), these branch frameworks accept feature output from the original decoder as input for skip connections.

PatchGAN [25], the discriminator block, consists of convolutional layers, normalization layers, and Leaky ReLU activation layers. Normalization layers [46] are applied after each convolution to stabilize training, followed by Leaky ReLU activations [47] with a negative slope of 0.2 to introduce non-linearity. This structure enables the discriminator to evaluate local patches of the input image, focusing on fine-grained details to effectively distinguish between real and generated images for FI and FFA.

### 2.3 Auxiliary Learning

Auxiliary learning improves model training by introducing additional, related tasks alongside the primary task. These secondary objectives help the model learn more generalized features, improving performance and generalization. It has been proven to effectively enhance model performance on the main task, guiding subsequent model construction to consider establishing related auxiliary tasks [48, 49]. In this study, we designate the generation of image structures as the auxiliary task and the generation of fluorescein angiography as the primary task. Inspired by the potential performance improvement of the main task through increased model depth with additional auxiliary tasks [50], in each training epoch, the branch model was used as deep extensions of the current model.

### 2.4 Structure Self-supervised Representation Learning

Self-supervised representation learning enables models to learn useful data features without labeled data by generating supervisory signals from the input itself. It captures meaningful structures that can be used for tasks like classification or generation and is particularly useful in fields where labeled data is scarce [51]. Self-supervised representation learning has made significant progress in the field of image processing [52-54], and similar concepts have shown good results in fundus image processing [55, 56].

Correctly identifying the content information of the original image and preserving it within the model is a crucial part of the generation process. Existing research indicates that without focusing on the structural information of fundus images, the reconstructed images may contain numerous meaningless capillaries [57]. We proposed a structure self-supervised representation, using the high-frequency information of the image itself as a self-supervised constraint during the training process can effectively address the issue of incomplete content in fundus images during translation [57, 58].

The structural images were generated as labels to supervise the model training. By reading the input image and extracting its green channel, a mask is then generated and applied with Gaussian filtering (with a kernel size of 5x5) to smooth the image. Histogram equalization and adaptive histogram equalization [59] are performed to enhance image contrast, using a tile size of 10x10 for adaptive equalization, followed by homomorphic filtering [60] to equalize illumination. Gamma correction [61] is applied with a gamma value of 1.5 to further enhance details. A filter is then constructed to process the gamma-corrected image, with a width of 1 and a length of 10 pixels, and the mask is applied to the processed image. Finally, the dynamic range of the image is adjusted through grayscale stretching, and Otsu's method is used for binarization, resulting in an enhanced binary image. The structure extraction algorithm for FFA includes an additional step of pixel value inversion.

### 2.5 Cycle Learning

Cycle learning can help models learn effective translation relationships without strict alignment (registration) [62-64]. It typically involves two main components: a forward cycle and a backward cycle. The forward cycle refers to translating an image from the source domain to the target domain, and the backward cycle involves translating the generated target image back to the source domain. The goal is to ensure that the generated image retains the content and structure of the original image from the source domain after both transformations. We incorporated the concept of cycle learning and structure self-supervised representation learning, guiding the model to simultaneously focus on cross-domain translation and the preservation of image structure. A branch model was used to extend the branching tasks, enabling cyclical image generation. The same training process is applied to both FI and FFA. Unlike cycle consistency [65], for intermediate results such as FIS from the model outputs, besides the discriminator loss, the norm loss can also be calculated.

### 2.6 Objective Function

The input image is processed by the model to output the image structure. For the loss function of the structure image, the first-order norm is uniformly used. The structural losses for FI and FFA are as follows (eqs **1**, **2**):

**Table:** 

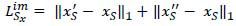	(1)

**Table:** 

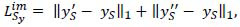	(2)

where 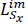 represents the structural loss of FI, 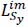 represents the structural loss of FFA. This loss function helps ensure that the image structure remains as consistent as possible throughout the training process.

FI and FFA generation loss also employs norm loss, with weight coefficients used to adjust the importance of each loss (eqs **3**, **4**):

**Table:** 

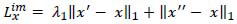	(3)

**Table:** 

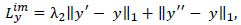	(4)

where 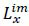 and 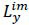 represent the FI and FFA image generation losses, respectively, with *λ*_1_ = 10, *λ*_2_ = 10. This loss function provides explicit labels to the model, helping to prevent training failure.

The adversarial loss is a critical component of GANs, where the generator creates fake data and the discriminator distinguishes it from real data. The generator is penalized when the discriminator successfully identifies fake data, motivating the generator to improve and produce more realistic outputs. This competition drives both networks to enhance their performance, resulting in high-quality synthetic data.

In this study, adversarial loss is uniformly calculated using the method proposed by LSGAN [66]. We employ four discriminators to separately evaluate FI, FFA, and their corresponding structure:

**Table:** 

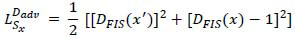	(5)

**Table:** 

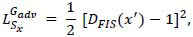	(6)

where 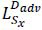, 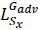 represent the adversarial losses for the discriminator and generator of the FIS. The adversarial loss for images is calculated using a method similar to that described in (Eqs **5** and **6**).

## 3 EXPERIMENTAL

### 3.1 Datasets

#### 3.1.1 Private Dataset (PDS)

We obtained FI and FFA from 331 patients with diabetic retinopathy, selecting 600 pairs of 768 × 768 images to form the usable dataset. In supervised learning, the FFA generated by translating the FI should ideally correspond pixel-by-pixel with the actual images to achieve optimal training results. However, such datasets are challenging to obtain in practice due to involuntary eye movement [67]. In real-world conditions, acquiring paired FI and FFA under the same conditions from the same patient is difficult, with many image pairs exhibiting displacement.

In this study, we defined the concept of pseudo-paired images. After manually selecting similar FI and FFA, the optic disc positions of a 512 × 512 FI and its corresponding FFA should be within ± 1 cm of each other. Using this method, we selected 433 pairs of data, with 347 pairs used as the training set and 86 pairs as the test set.

#### 3.1.2 PDS-c

The quality of FI or FFA can be degraded by various factors during the imaging process. In this study, we observed that the overall contrast of the acquired images was relatively low. Therefore, we applied Contrast Limited Adaptive Histogram Equalization (CLAHE) [68] to enhance the contrast of the training data. The enhanced image results are shown in Fig. (**2**). Similarly, we used the same random seed to partition the dataset, with 80% of the image pairs used as the training set.

#### 3.1.3 PDS-r

The acquired image pairs are not strictly aligned, which hinders the establishment of precise pixel-level constraints for guiding model generation in supervised learning. In this study, we used the Insight Segmentation and Registration Toolkit (ITK) [69] to register FI and FFA, resulting in the registration dataset through rigid registration [70, 71]. Similarly, we partitioned this dataset using the same method.

#### 3.1.4 PDS-c-r

After applying CLAHE to the PDS dataset, we used the ITK tool for rigid registration, then split the dataset into training and test sets.

#### 3.1.5 F&C

(Fundus Fluorescein Angiogram Photographs & Color Fundus Images of Diabetic Patients): In line with previous work, we acquired an additional private dataset, which includes 30 pairs of FI and pseudo-paired FFA from healthy individuals, and 30 pairs of pseudo-paired images from patients with diabetic retinopathy. The image scale is 720 × 576. Considering the complexity of the task and the scale of images required for model learning, this dataset is not used in the model training process but is only utilized as a cross-domain test set to evaluate the model's generalization ability.

### 3.2 Setup

CUDA Version: 12.0, NVIDIA Driver Version: 525.89.02. A single NVIDIA 2080 Ti GPU was used for model training and testing. The model has 89.283 million parameters, with an average memory usage of 3583 MiB during training. The image scale was adjusted to 286 × 286 for training and resized to 256 × 256 after data augmentation to support rapid training and testing. Approximately 1.78 hours were required to complete 200 epochs of training. For the image generation task, the training batch size was set to 3, instance normalization was chosen, and RMSProp [72] was used as the optimizer. The learning rate was set to 2e-4, with a linear decay starting after 150 epochs.

## 4 RESULTS

### 4.1 Comparative Experiment

In previous research, the limited data scale led to an overemphasis on model performance on the validation set, with inadequate attention given to evaluation on the test set [38-40]. In practical applications, image quality is influenced by multiple factors, and real images often fall outside the domain of the current model. Therefore, constructing a multi-source test dataset to evaluate the model's general performance is necessary.

In this study, we randomly selected images from the 347 pairs in the training set for validation and tested the model's training effect on 86 pairs of unseen images. Additionally, we used 60 pairs of unseen images from the F&C dataset to test the inference model's actual performance in cross-domain tasks, using FID [73] and KID as model performance evaluation metrics. The lower their values, the closer the generated images are to the target.

We used the publicly available code on GitHub and the recommended training configurations as comparisons. It is important to note that models like UWAT-GAN and UWAFA-GAN cannot be directly applied to this experiment and require adjustments to the image size and related code.

As illustrated in Fig. (**1**), our model attains the top performance in both FID and KID metrics. For the model incorporating feature loss (VGG loss [74]), such as VTGAN [40] and UWAT-GAN [42], CLAHE data augmentation or ITK rigid registration methods did not significantly improve its performance on the test set PDS-r. In contrast, models that do not rely on pixel-level constraints, such as StillGAN, are more sensitive to the image's inherent contrast. After applying CLAHE data augmentation to the original data, the performance on the test set PDS-c significantly improves.

Fig. (**3**) presents the visual outcomes of the model on the PDS-c-r validation set. In column one, the FFA image displays white spot noise in the macular region. Our model effectively resolved this issue. StillGAN exhibited inaccuracies in choroidal reconstruction, and Pix2Pix failed to accurately interpret image content in the validation set. Similarly, VTGAN and UWAT-GAN did not effectively avoid the noise problem. In column two, FI contains artifacts. Although our model did not introduce artifacts, it showed reconstruction errors at the artifact edges. StillGAN retained the artifacts in the FFA images Table **1**.

VTGAN and UWAT-GAN were not influenced by the FI artifacts, but due to rigid registration displacing the FFA labels, their reconstructed FFA images did not reference the FI outline correctly. In column four, FFA shows fluorescein leakage, indicating an underlying eye condition. This scenario requires high reconstruction capability from the model. Even models like VTGAN and UWAFA-GAN, which focus on FFA results, could not reconstruct this situation accurately.

The model comparison on the PDS-c-r is shown in Fig. (**4**). In column one, our model references the original image structure during generation, resulting in the replacement of the original artifact areas with corresponding artifacts or capillaries. StillGAN, VTGAN, and UWAT-GAN fail to generate images that align with the original semantics effectively. In column two, only our model considered the original structure of the macular region during reconstruction and generated the corresponding area appropriately. It is noteworthy that even when trained on a dataset with contrast enhancement and rigid registration, Pix2Pix, VTGAN, and UWAT-GAN still failed to generate effectively on the test set. This indicates that in this translation task, when the dataset scale increases, models that rely on directional pixel-level constraints to guide the training process cannot adequately fit the errors caused by flexible registration [75] and content differences between image pairs.

Further testing of the model's capability was conducted on the F&C dataset. The F&C dataset consists of high-quality images with low degrees of retinal disease in patients, and we consider this dataset to belong to a different image domain from the training dataset. As shown in Table (**2**), among the four pre-trained models, three performed best on the F&C test set. The results indicate that even for cross-domain tasks, our model still shows a significant advantage.

### 4.2 Ablation Experiment

The effectiveness of the proposed modules is evaluated through an ablation study. The Cycle Learning (CL) module indicates whether the model incorporates the FFA-to-FI generation framework. Additionally, the Auxiliary Learning method is integrated with the Structure Self-Supervised Representation Learning (SSM) module for joint assessment, representing whether the model employs a branch model and corresponding loss functions to enhance model depth. As shown in Table (**3**), using the same training configuration for each row and evaluating the results on the same test sets, the results demonstrate that incorporating the full module improves the FID and KID scores.

### 4.3 Expert Experiment

In this study, we invited three experts, including ophthalmologists, to assess the authenticity of the images generated by the model. Each expert received a set of 50 mixed true and false images, without being informed of their labels. The final evaluation employed a majority voting method. As shown in Table (**4**), the results revealed that the accuracy of the synthetic images was only 30%, with a recall rate of 47%, indicating that at least 50% of the synthetic images could be considered as real images.

## 5 DISCUSSION

While our model demonstrates advantages in evaluation metrics on the PDS series dataset and F&C dataset, several challenges remain in generating reliable images. As illustrated in the third column of Fig. (**4**), the model takes into account the existing illumination conditions of the FI and incorporates pathological features learned from the dataset images when reconstructing vascular structures. The generated FFA images have a lighting pattern that mirrors the distribution of the original FA images. However, in authentic FFA images, the illumination is influenced by several factors, including the timing of image capture and the effects of fluorescein, which our current model’s output lacks. Our methods do not fully mimic the complex dynamics seen in real clinical FFA images.

Furthermore, it was observed that real FI and FFA images are not perfectly aligned, even after applying ITK rigid registration, as depicted in Fig. (**2**). This misalignment poses a significant challenge for models relying on pixel-level losses (*e.g*., L1 or L2 norms), such as Pix2Pix [25] and Fundus2Angio [38]. These methods struggle to achieve satisfactory results in this case, as the inherent differences between real FI and FFA images cannot be fully captured by simple pixel-based comparison.

However, methods that focus solely on feature-level losses, such as Attention2Angio [39] and CutGAN [27], also failed to deliver optimal performance in our experiments. These approaches are designed to capture structural features, but they may overlook subtle yet crucial details related to the illumination and alignment issues present in fundus images.

Combining both pixel-level and feature-level losses has shown promising results in our experiments. However, optimizing the balance between these losses remains a critical challenge. Fine-tuning the weights of these loss functions, and ensuring they work in harmony, is an essential area for future work.

Medical imaging prioritizes authenticity over artistic interpretation, making accuracy essential. A fundamental information gap exists between FI and FFA. While synthesizing FFA-style images from FI is feasible, the generated images retain only the information present in FI and lack critical FFA-specific features, such as capillary non-perfusion areas. This limitation raises concerns about the direct clinical applicability of synthesized FFA images. A key research challenge moving forward is to enhance FFA generation by integrating FI data with additional auxiliary information. Bridging the information gap between FI and FFA will be crucial for improving the realism and clinical reliability of these synthesized images.

## CONCLUSION

This study proposes a generative adversarial network model for translating fundus images (FI) into fundus fluorescein angiography (FFA) using clinical datasets. By incorporating a structure self-supervised representation cycle learning method, the model effectively guides the cycle training process, enabling accurate and high-fidelity image translation. This approach addresses the limitations of small datasets encountered in previous studies and demonstrates superior generalization capabilities across diverse clinical samples.

Experimental results confirm that the proposed model not only preserves anatomical structures but also enhances the representation of vascular and pathological features in the generated FFA images. This advancement holds significant implications for ophthalmology, as it facilitates non-invasive visualization of retinal circulation, potentially reducing the need for invasive fluorescein angiography procedures. Furthermore, the ability to generate high-quality synthetic FFA images supports automated disease screening, early diagnosis, and disease progression monitoring, thereby improving clinical decision-making and patient management.

Future research will focus on refining the model by integrating additional multi-modal data and exploring more advanced generative learning techniques to enhance robustness and interpretability. Moreover, large-scale prospective studies will be conducted in collaboration with ophthalmologists to assess the model’s clinical applicability and effectiveness in real-world diagnostic workflows. Ultimately, this work contributes to bridging the gap between artificial intelligence and clinical practice, expanding the role of deep learning in medical imaging and disease diagnosis.

## AUTHORS’ CONTRIBUTIONS

The authors confirm their contribution to the paper as follows: X.W.: Study conception and design; Y.C., D.G.: Data collection; C.L., Q.L., Q.Y.: Draft manuscript; R.M.: Validation. All authors reviewed the results and approved the final version of the manuscript.

## Figures and Tables

**Fig. (1):**
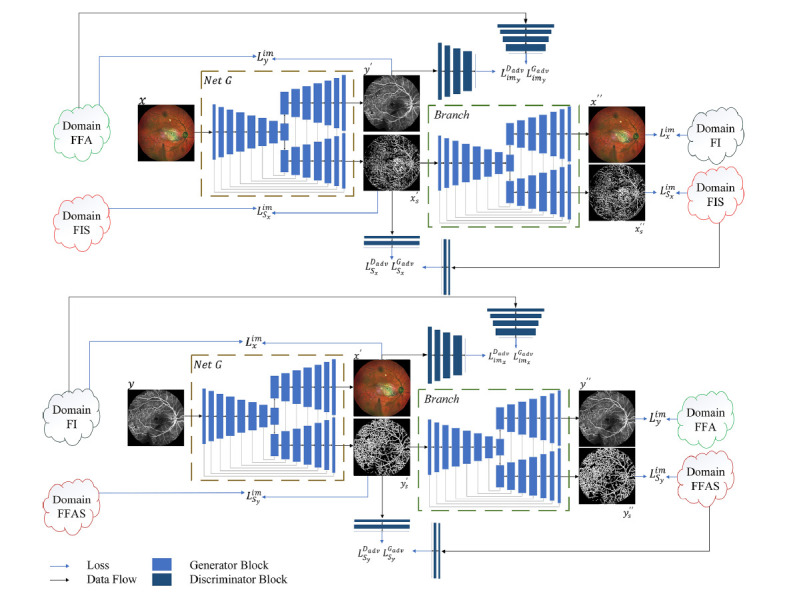
The two-stage training process for translating fundus images (FI) into fundus fluorescein angiography (FFA) images. FI-to-FFA translation (top section of the figure): Input the FI *x* to generate the FFA *y*’, then calculate the difference between *y*’and the ground truth *y*. Maintain the structural information of the FI unchanged, cyclically generate *x*” through *x*’*_s_*. FFA-to-FI translation (bottom section of the figure): similar to FI-to-FFA, with the input image replaced by FFA.

**Fig. (2):**
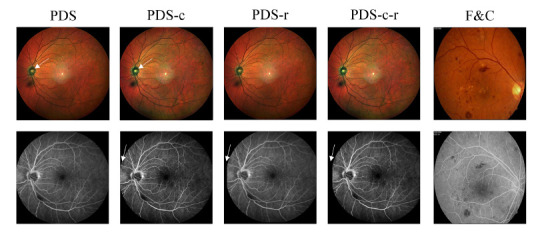
The five datasets used in the experiment. PDS-c enhances the PDS image dataset using CLAHE, PDS-r enhances the PDS dataset with strict registration, and F&C serves as a public test benchmark.

**Fig. (3):**
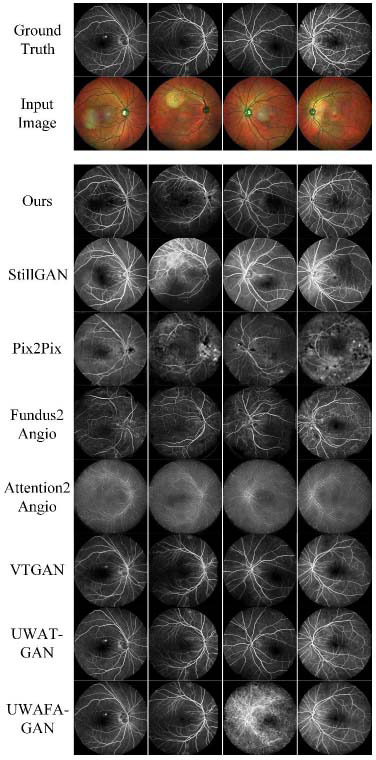
Performance on the PDS-c-r validation dataset. Previous methods did not perform well in these experiments when following the publicly recommended training configurations.

**Fig. (4):**
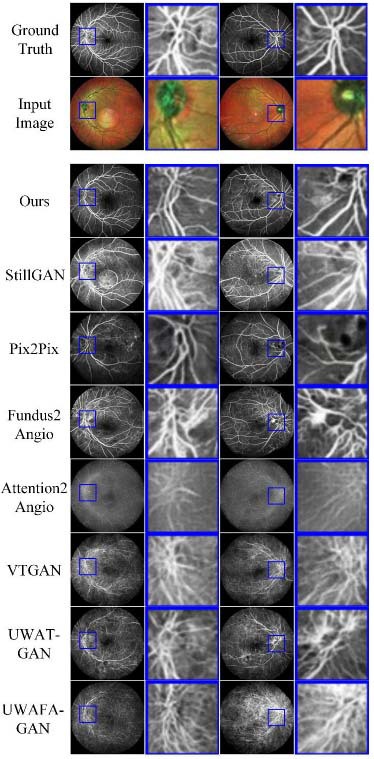
Performance on the PDS-c-r test dataset. Compared to other methods, our generated images exhibit higher visual perceptual quality.

**Table 1:** Performance on the PDS series datasets.

**Frchet Inception Distance (FID)**				
**Model**	**PDS**	**PDS-C**	**PDS-R**	**PDS-C-R**
StillGAN	166.2725	67.2198	124.8765	62.7345
Pix2Pix	80.9649	67.4209	109.5176	78.8366
Fundus2Angio	80.0247	113.4379	107.5590	96.7844
Attention2Angio	128.2377	138.4945	148.8163	165.8633
VTGAN	105.0893	95.1812	94.3646	111.2940
UWAT-GAN	82.2145	81.9875	74.5649	86.7143
UWAFA-GAN	95.7990	104.6735	80.2913	109.8889
**Ours**	**67.7801**	**60.2757**	**64.8326**	**55.6429**
**Kernel Inception Distance (KID)**				
**Model**	**PDS**	**PDS-C**	**PDS-R**	**PDS-C-R**
StillGAN	0.1645	0.0478	0.0990	0.0378
Pix2Pix	0.0588	0.0444	0.1068	0.0533
Fundus2Angio	0.0648	0.1108	0.1232	0.0915
Attention2Angio	0.1726	0.1868	0.2105	0.2345
VTGAN	0.1122	0.0929	0.0983	0.1165
UWAT-GAN	0.0777	0.0694	0.0655	0.0764
UWAFA-GAN	0.1006	0.1141	0.0749	0.1151
**Ours**	**0.0481**	**0.0348**	**0.0443**	**0.0253**

**Table 2:** Generalization performance on the F&C dataset (after training on the PDS series datasets).

**Frchet Inception Distance (FID)**				
**Model**	**PDS**	**PDS-C**	**PDS-R**	**PDS-C-R**
StillGAN	171.2624	111.4695	135.9430	105.0730
Pix2Pix	140.9799	119.4406	129.7031	144.2932
Fundus2Angio	134.8456	182.7002	172.3457	156.0158
Attention2Angio	122.0610	93.5541	129.4543	172.5857
VTGAN	167.6318	139.9864	156.2156	183.0476
UWAT-GAN	162.2778	169.5467	154.7113	161.5885
UWAFA-GAN	169.3952	167.4788	154.7113	183.8479
**Ours**	**123.5146**	**99.6492**	**103.6025**	109.7464
**Kernel Inception Distance (KID)**				
**Model**	**PDS**	**PDS-C**	**PDS-R**	**PDS-C-R**
StillGAN	0.1924	0.1000	0.1260	0.0983
Pix2Pix	0.1463	0.1183	0.1281	0.1530
Fundus2Angio	0.1533	0.2256	0.1871	0.1805
Attention2Angio	0.1540	0.0871	0.1612	0.2300
VTGAN	0.2079	0.1636	0.1967	0.2310
UWAT-GAN	0.1990	0.2165	0.1840	0.1882
UWAFA-GAN	0.1990	0.2165	0.1840	0.2280
**Ours**	**0.1458**	**0.0993**	**0.1045**	0.1096

**Table 3:** Effectiveness of self-supervised representation learning and cycle learning.

**Module**	**FID**	**KID**
Ours w.o. SSM, CL	66.5456	0.0406
Ours w.o. CL	61.4269	0.0323
Ours	55.6429	0.0253

**Table 4:** Evaluation through expert majority voting.

	**Precision**	**Recall**	**F1-score**
**False**	0.32	0.47	0.38
**True**	0.64	0.48	0.55

## Data Availability

The data that support the findings of this study are available from the corresponding authors [Q.Y] and [Q.L] upon reasonable request.

## LIST OF ABBREVIATIONS

GAN: Generative Adversarial Network
FIS: Fundus Image’s Structure
FFAS: Fundus Fluorescein Angiography Structure (domain)
FFA: Fundus Fluorescein Angiography
FI: Fundus Image
